# Association of dietary live microbe intake with diabetic kidney disease in patients with type 2 diabetes mellitus in US adults: a cross-sectional study of NHANES 1999–2018

**DOI:** 10.1007/s00592-023-02231-8

**Published:** 2024-02-24

**Authors:** Min Wang, Zhao-hui Huang, Yong-hong Zhu, Shuai Li, Xin Li, He Sun, Ping He, Ya-li Peng, Qiu-Ling Fan

**Affiliations:** 1 Department of Nephrology, First Hospital of China Medical University, Shenyang, Liaoning China; 2 Department of Nephrology, The second affiliated Hospital of Guangzhou Medical University, Guangzhou, Guangdong China; 3 Department of Respiratory and Critical Care Medicine, The First Hospital of Anhui Medical University, Hefei, Anhui China; 4 Department of Endocrinology, Shengjing Hospital of China Medical University, Shenyang, Liaoning China; 5 Department of Nephrology, Shanghai General Hospital, Shanghai Jiao Tong University School of Medicine, Shanghai, China; 6 Department of Nephrology, The Fourth Hospital of China Medical University, Shenyang, Liaoning China

**Keywords:** Dietary live microbe, Type 2 diabetes mellitus, Diabetic kidney disease, Cross-sectional study, NHANES

## Abstract

**Aims:**

Several studies have reported dietary microorganisms’ beneficial effects on human health. We aimed to detect the potential association between dietary live microbe intake and diabetic kidney disease (DKD) in patients with type 2 diabetes mellitus (T2DM) through a cross-sectional analysis of the National Health and Nutrition Examination Survey from 1999 to 2018.

**Methods:**

According to the Sanders classification system of dietary live microbes, the study participants were divided into three groups: low, medium, and high live microbe groups. In patients with T2DM, DKD was assessed by glomerular filtration rate (< 60 mL/min/1.73 m^2^ using the Chronic Kidney Disease Epidemiology Collaboration algorithm), proteinuria (urinary albumin to creatinine ratio ≥ 30 mg/g), or both. Weighted univariate and multivariate logistic regression and subgroup analyses were conducted to investigate the independent association between dietary live microbe and DKD.

**Results:**

The study included 3836 participants, of whom 1467 (38.24%) had DKD for the diagnosis. Our study demonstrated that participants in the high dietary live microbe group were more likely to be older, female, non-Hispanic White, have higher education levels, have a lower prevalence of smoking, have a high poverty-income ratio, have higher energy intake, lower haemoglobin (HbA1c) and serum creatinine levels, and lower risk of progression. After adjustment for covariates, patients in the high dietary live microbe group had a low prevalence of DKD, whereas no significant association with DKD was found between the medium and low dietary live microbe groups. No statistically significant interaction was observed in all subgroup analyses except for HbA1c (*p* for interaction < 0.05).

**Conclusions:**

Our results indicate that high dietary live microbe intake was associated with a low DKD prevalence.

## Introduction

Type 2 diabetes mellitus (T2DM) has become a global health problem with increasing incidence worldwide. It is estimated that 537 million adults worldwide will face this problem in 2021 [1]. Despite the advancements in treatment with sodium-glucose cotransporter 2 inhibitors and glucagon-like peptide-1 receptor agonists [2–4], patients with T2DM remain at risk of developing diabetic kidney disease (DKD), which is one of the top 10 causes of death globally [5]. Therefore, exploring new approaches for preventing the onset and progression of DKD is vital.

Despite the obvious positive impact of more hygienic food on public health, reduced microbial exposure may also have unintended adverse health effects. The 'hygiene hypothesis' reveals a potential role for microbes, whereby lack of exposure to microorganisms triggers immune dysregulation, leading to an increase in chronic inflammatory diseases [6]. S Several studies have shown that live microbes in the diet benefit human health [7, 8]. Live and safe microbes in the diet can reach the gut via contact with the digestive tract’s mucosal surface, regulating the systemic immune response, enhancing intestinal function, and weakening susceptibility to chronic disease [9]. Growing evidence supports an important role for the gut microbiota and its metabolites in the pathophysiologic processes of DKD [10–12]. A study showed that a significant decrease in the abundance of *Rothia* and *Faecalibacterium prausnitzi* in the gut microbiota was associated with insulin resistance in European women with T2DM [13]. Improving gut microbiota dysbiosis through *Bifidobacterium* supplementation was found to correct impaired glucose tolerance and attenuate insulin resistance in diabetic mice [14]. By regulating gut microbiota, enriching short-chain fatty acids (SCFAs)-producing bacteria and increasing SCFAs production, dietary fiber shows novel nephroprotective effects in reducing albuminuria, glomerular hypertrophy, capsular injury and renal interstitial fibrosis [15]. Gut dysbiosis may allow endotoxins and pathogens to cross the intestinal barrier, causing inflammation and oxidative stress, further accelerating renal injury [16]. In addition, the gut microbial dysbiosis may lead to alterations in microbial metabolites. For example, excess acetate produced by gut dysbiosis has been shown to be involved in renal injury through activation of the intrarenal RAS and to contribute to tubulointerstitial injury by modulating cholesterol homeostasis in vivo and in vitro [10, 17]. In view of the potential pathogenic role of gut dysbiosis in DKD, it is suggested that the treatment of DKD may be facilitated by intervening in the gut microbiota.

The Sanders classification system categorizes foods into low (< 10^4^ CFU/g), medium (10^4^–10^7^ CFU/g), and high (> 10^7^ CFU/g) levels of live microbes based on the number of living microorganisms in the diet based on four experts in the field. Dairy products and other foods have been associated with the incidence of chronic kidney disease (CKD) [18–20]. However, the relationship between dietary live microbe intake and DKD has not been fully elucidated. Therefore, this study aims to investigate the potential association between dietary live microbe intake and DKD in adults with T2DM using the 1999–2018 data from the National Health and Nutrition Examination Survey (NHANES) based on Sanders’ dietary live microbe’ classification system.

## Methods

### Study population

**Fig. 1:**
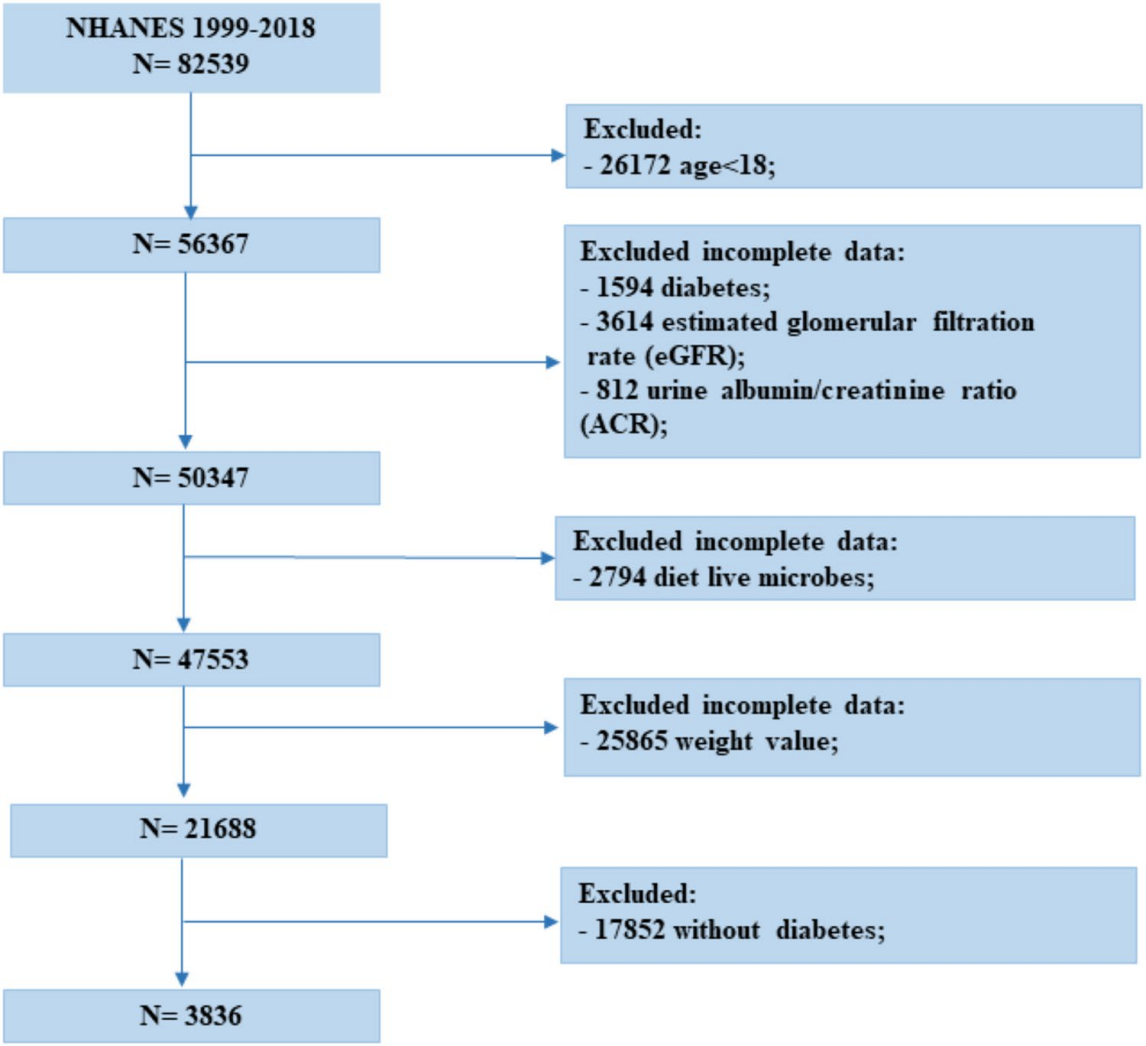
A flowchart showing the selection of study participants

Data for this study were extracted from the NHANES (1999–2018), a representative cross-sectional survey conducted by the National Center for Health Statistics and the Centers for Disease Control and Prevention. The survey samples the US population using a stratified, multi-stage probability approach and provides health and nutrition statistics for non-institutionalized civilians in the United States. Detailed statistics are accessible at https://www.cdc.gov/nchs/nhanes/. Participants under the age of 18 years or with missing values for important variables were excluded. The final sample size for this study was 3836 participants (Fig. 1).

### Dietary intakes and live microbial category

In NHANES, dietary intake data were collected through 24-h dietary recall interviews conducted at mobile screening centers. The complete description of the NHANES dietary survey methodology is available on the web [21]. The University of Texas Food Intake Analysis System and the USDA Survey Nutrient Database were used to assess nutrient and energy intake. To determine the estimated number of live microorganisms per gram of food, four experts in the field assessed the 9388 food codes contained in the 48 subgroups of the NHANES database. Based on this estimate, foods were classified into three categories: low, medium, or high levels of live microorganisms. Foods with < 10^4^ CFU/g, 10^4^–10^7^ CFU/g, and > 10^7^ CFU/g were classified as low, medium, and high, respectively. These categories included pasteurized foods (< 10^4^ CFU/g), unpeeled fresh fruits and vegetables (10^4^–10^7^ CFU/g), and unpasteurized fermented foods and probiotic supplements (> 10^7^ CFU/g). This categorization aligns with previous literature [20].

### Outcome variable

Diabetes was defined by the following criteria: (1) a previously reported diagnosis by medical professionals, (2) fasting plasma glucose ≥ 7.0 mmol/L, (3) glycosylated hemoglobin (HbA1c) ≥ 6.5%, (4) 2-h OGTT blood glucose (mmol/l) ≥ 11.1, or (5) use of diabetes medication or insulin [22]. The urine albumin/creatinine ratio (ACR) was used to compute the ACR (< 30, A1; 30–300, A2; > 300 mg/g, A3). The estimated glomerular filtration rate (eGFR) scores were calculated using the Chronic Kidney Disease Epidemiology Collaboration algorithm, which is divided into G1 (90 mL/min/1.73 m^2^), G2 (60–89 mL/min/1.73 m^2^), G3a (45–59 mL/min/1.73 m^2^), G3b (30–44 mL/min/1.73 m^2^), G4 (15–29 mL/min/1.73 m^2^), and G5 (< 15 mL/min/1.73 m^2^) [23]. DKD was diagnosed in patients with T2DM when their ACR was ≥ 30 mg/g and/or their eGFR was < 60 mL/min/1.73 m^2^. Patients were classified into moderate risk (G3a and A1 or G1–G2 and A2), high risk (G3b and A1, or G3a and A2, or G1–G2 and A3), and very high risk (G4–G5, G3b, and A2–A3 or G3a and A3) [24].

### Covariates

Participants’ information including age, sex (male and female), race (non-Hispanic Whites, non-Hispanic Blacks, Mexican Americans, and others), body mass index (BMI, kg/m^2^), education level (< high school, high school and > high school), poverty-income ratio (PIR), smoking status (never/former/now), high-density lipoprotein cholesterol (HDL-C), low-density lipoprotein cholesterol (LDL-C), total cholesterol (TC), triglycerides (TG), alanine aminotransferase(ALT), aspartate aminotransferase (AST), serum creatinine (Cr), uric acid (UA), total energy, hypertension (yes and no), metabolic syndrome (yes and no), and cardiovascular disease (CVD, yes and no) were collected.

### Statistical analysis

Appropriate examination weights were used to represent the complex survey design. Continuous variables were presented as mean ± standard deviation (SD), and comparisons between two groups were conducted using the independent samples T-test (normal distribution) or Mann–Whitney U test (skewed distribution), respectively. Categorical variables were displayed as numbers (weighted percentage) and compared using the χ^2^ test. The study participants were divided into three groups based on the food code assignments of low, medium, and high: low dietary microbe group (all foods are low); medium dietary microbe group (any food is medium but not high); and high dietary microbe group (any food is high). Weighted multivariate logistic regression analysis was used to evaluate the correlation between dietary live microbe intake and DKD prevalence in different models. The crude model was not adjusted for any potential confounding factors. Model 1 was adjusted for age, sex, and race. Model 2 was further adjusted for BMI. Model 3 added education, smoking status, PIR, UA, TG, TC, HDL-C, LDL-C, total energy, hypertension, CVD, and metabolic syndrome as covariates to Model 2. The association between dietary live microbe and DKD was further analyzed stratified by age (< 60/ ≥ 60 years), sex (male/female), race/ethnicity, education, PIR (< 1.3, 1.3–1.8, > 1.8), smoking status, BMI (< 25.0, 25.0–30.0, ≥ 30.0 kg/m^2^), HbA1c (< 7.0, ≥ 7.0%), hypertension (yes/no), CVD (yes/no) and Metabolic syndrome (yes/no). Missing values for existing examples of those variables were filled in using the mode for categorical variables or the median for continuous variables. An interaction term was also added to test for heterogeneity of association between subgroups. The statistical analysis was performed using R software version 4.2.2 (http://www.R-project.org, The R Foundation, Austria). The “nhanesR” package was used to extract and analyze the data and *P* values < 0.05 were considered statistically significant.

## Results

### Baseline characteristics of the included participants

A total of 3836 participants were included, of whom 1467 were diagnosed with DKD. Table 1 displays the weighted baseline characteristics of participants with or without DKD in this study. Overall, age, education, PIR, HbA1c, Cr, UA, TG, LDL-C, eGFR, UACR, total energy, hypertension, CVD, metabolic syndrome, and prognosis were statistically significant between participants with and without DKD. Moreover, participants with DKD were more likely to consume fewer dietary live microbes (*p* = 0.001).

**Table 1:** General characteristics of participants (n = 3836) with T2DM in the NHANES 1999–2018

Character	Total	Non-DKD	DKD	*P* value
Age(years)	58.83(0.34)	56.29(0.39)	63.62(0.48)	< 0.0001
Gender (%)				0.87
Female	1845(48.1)	1149(49.11)	696(48.74)	
Male	1991(51.9)	1220(50.89)	771(51.26)	
Race/ethnicity (%)				0.84
Non-Hispanic white	1505(39.23)	882(64.11)	623(64.12)	
Non-Hispanic black	847(22.08)	529(13.32)	318(13.61)	
Mexican American	782(20.39)	485(9.08)	297(9.55)	
Other race	702(18.3)	473(13.50)	229(12.73)	
Education (%)				< 0.001
< High school	1356(35.35)	774(21.53)	582(29.17)	
High school	894(23.31)	555(26.22)	339(27.42)	
> High school	1586(41.35)	1040(52.25)	546(43.42)	
PIR	2.68(0.04)	2.80(0.05)	2.46(0.06)	< 0.0001
Smoking status (%)				0.25
Never	1915(49.92)	1215(50.24)	700(47.02)	
Former	1288(33.58)	755(32.72)	533(36.37)	
Now	633(16.5)	399(17.04)	234(16.61)	
BMI (kg/m^2^)	32.54(0.17)	32.67(0.22)	32.29(0.27)	0.27
HbA1c (%)	6.99(0.04)	6.85(0.04)	7.27(0.06)	< 0.0001
Cr (μmol/L)	82.19(0.85)	72.55(0.45)	100.31(2.29)	< 0.0001
UA (μmol/L)	347.92(2.16)	334.66(2.40)	372.87(3.34)	< 0.0001
TG(mmol/L)	1.95(0.04)	1.86(0.04)	2.13(0.08)	0.002
TC(mmol/L)	4.91(0.03)	4.92(0.03)	4.89(0.04)	0.6
HDL-C(mmol/L)	1.26(0.01)	1.25(0.01)	1.28(0.02)	0.19
LDL-C(mmol/L)	2.80(0.02)	2.84(0.03)	2.73(0.03)	0.01
eGFR (mL/min/1.73 m^2^)	85.57(0.50)	92.60(0.50)	72.33(0.93)	< 0.0001
UACR (mg/g)	117.21(11.15)	10.02( 0.16)	318.90(30.26)	< 0.0001
Total energy (kcal)	1929.25(17.65)	1989.45(23.39)	1815.96(25.29)	< 0.0001
Hypertension				< 0.0001
No	1127(29.38)	856(37.21)	271(19.68)	
Yes	2709(70.62)	1513(62.79)	1196(80.32)	
CVD (%)				< 0.0001
No	2927(76.3)	1950(82.53)	977(67.34)	
Yes	909(23.7)	419(17.47)	490(32.66)	
Metabolic syndrome (%)				0.001
No	950(24.77)	651(24.99)	299(18.95)	
Yes	2886(75.23)	1718(75.01)	1168(81.05)	
Prognosis				< 0.0001
Low risk	2369(61.76)	2369(100.00)	0( 0.00)	
Moderate risk	899(23.44)	0( 0.00)	899(63.61)	
High risk	331(8.63)	0( 0.00)	331(21.92)	
Very high risk	237(6.18)	0( 0.00)	237(14.47)	
Dietary live microbe group (%)				0.001
Low	1410(36.76)	843(32.24)	567(38.03)	
Medium	1732(45.15)	1070(44.82)	662(44.95)	
High	694(18.09)	456(22.94)	238(17.02)	

Data are presented as frequencies (percentages) or mean (SD)

*DKD* diabetic kidney disease, *T2DM* type 2 diabetes mellitus, *PIR* poverty-income- ratio, *BMI* the body-mass index is determined as follows: the weight in kilograms (Kgs)/(height in square meters (m^2^), *HbA1c* haemoglobin, *Cr* serum creatinine, *UA* serum uric acid, *TG* triglycerides, *TC* total cholesterol, *HDL-C* high-density lipoprotein cholesterol, *LDL-C* low-density lipoprotein cholesterol, *eGFR* estimated glomerular filtration, *UACR* urinary albumin/creatinine ratio, *CVD* cardiovascular diseases

### Participants in different dietary live microbe groups

The total weighted prevalence of DKD was 38.53%, 34.77%, and 28.28% in the low, medium, and high dietary live microbial groups, respectively. The characteristics of the participants in the three groups are shown in Table 2. Participants in the high dietary live microbe group were more likely to be older, female, non-Hispanic White, more educated, less smoking, have high PIR, have higher energy intake, have lower HbA1c and Cr levels, and have lower risk of progression (all *p* < 0.05). In contrast, no significant trends were observed for BMI, UA, TG, TC, LDL-C, eGFR, UACR, hypertension, CVD, and metabolic syndrome.

**Table 2:** Participants’ clinical characteristics according to the different dietary live microbe groups

Character	total	Low	Medium	High	*P* value
Age (years)	58.83(0.34)	57.69(0.53)	59.97(0.47)	58.26(0.73)	0.003
Gender (%)					0.01
Female	1845(48.1)	649(45.77)	820(48.46)	376(55.37)	
Male	1991(51.9)	761(54.23)	912(51.54)	318(44.63)	
Race/ethnicity (%)					< 0.0001
Non-Hispanic white	1505(39.23)	508(59.62)	684(64.86)	313(69.87)	
Non-Hispanic black	847(22.08)	398(18.28)	344(11.98)	105( 8.54)	
Mexican American	782(20.39)	238( 8.29)	414(10.57)	130( 7.94)	
Other race	702(18.3)	266(13.81)	290(12.58)	146(13.65)	
Education (%)					< 0.0001
< High school	1356(35.35)	543(28.47)	617(23.67)	196(18.24)	
High school	894(23.31)	348(28.67)	388(26.57)	158(23.43)	
> High school	1586(41.35)	519(42.86)	727(49.76)	340(58.34)	
PIR	2.68(0.04)	2.43(0.06)	2.78(0.06)	2.88(0.08)	< 0.0001
Smoking status (%)					< 0.0001
Never	1915(49.92)	354(47.95)	694(48.82)	867(49.90)	
Former	1288(33.58)	254(40.46)	416(28.63)	618(35.07)	
Now	633(16.5)	86(11.59)	300(22.55)	247(15.03)	
BMI (kg/m^2^)	32.54(0.17)	32.81(0.30)	32.31(0.23)	32.57(0.37)	0.42
HbA1c (%)	6.99(0.04)	7.11(0.06)	6.96(0.05)	6.89(0.07)	0.04
Cr (μmol/L)	82.19(0.85)	84.32(1.42)	82.87(1.44)	77.23(1.08)	< 0.0001
UA (μmol/L)	347.92(2.16)	354.17(3.73)	345.56(2.96)	342.75(4.37)	0.07
TG (mmol/L)	1.95(0.04)	1.95(0.07)	1.96(0.06)	1.94(0.07)	0.98
TC (mmol/L)	4.91(0.03)	4.93(0.04)	4.89(0.04)	4.94(0.06)	0.63
HDL-C (mmol/L)	1.26(0.01)	1.25(0.01)	1.27(0.02)	1.27(0.02)	0.55
LDL-C (mmol/L)	2.80(0.02)	2.85(0.04)	2.77(0.03)	2.81(0.05)	0.21
eGFR (mL/min/1.73 m^2^)	85.57(0.50)	86.31(0.83)	84.36(0.73)	86.96(0.99)	0.09
UACR (mg/g)	117.21(11.15)	135.57(19.67)	119.39(16.62)	82.43(19.14)	0.13
Total energy(kcal)	1929.25(17.65)	1865.96(29.97)	1917.97(23.31)	2057.24(47.03)	0.01
Hypertension					0.59
No	1127(29.38)	411(32.38)	510(30.13)	206(31.22)	
Yes	2709(70.62)	999(67.62)	1222(69.87)	488(68.78)	
CVD (%)					0.15
No	2927(76.3)	1051(74.79)	1335(78.47)	541(78.73)	
Yes	909(23.7)	359(25.21)	397(21.53)	153(21.27)	
Metabolic syndrome (%)					0.27
No	950(24.77)	350(23.96)	448(23.42)	152(20.02)	
Yes	2886(75.23)	1060(76.04)	1284(76.58)	542(79.98)	
DKD					0.001
No	2369(61.76)	843(61.47)	1070(65.23)	456(71.72)	
Yes	1467(38.24)	567(38.53)	662(34.77)	238(28.28)	
DKD prognosis					0.02
Low risk	2369(61.76)	843(61.47)	1070(65.23)	456(71.72)	
Moderate risk	899(23.44)	340(23.74)	399(22.22)	160(19.01)	
High risk	331(8.63)	129(8.91)	157(7.65)	45(5.38)	
Very high risk	237(6.18)	98(5.88)	106(4.90)	33(3.89)	

Data are presented as frequencies (percentages) or mean (SD)

*DKD* diabetic kidney disease, *PIR* poverty-income- ratio, *BMI* the body-mass index is determined as follows: the weight in kilograms (Kgs)/(height in square meters (m^2^), *HbA1c* haemoglobin, *Cr* serum creatinine, *UA* serum uric acid, *TG* triglycerides, *TC* total cholesterol, *HDL-C* high-density lipoprotein cholesterol, *LDL-C* low-density lipoprotein cholesterol, *eGFR* estimated glomerular filtration, *UACR* urinary albumin/creatinine ratio, *CVD* cardiovascular diseases

### Association between different dietary live microbe and DKD

Table 3 lists the relationship between different dietary live microbe groups and DKD using univariate and multivariate weighted logistic analysis. Three models were constructed: the crude model (not adjusted for covariates), Model 1 (adjusted for age, sex, and race), Model 2 (adjusted for age, sex, race, and BMI), and Model 3 (added education, smoking status, PIR, UA, TG, TC, HDL-C, LDL-C, total energy, hypertension, CVD, and metabolic syndrome as covariates to Model 2). In the univariate logistic regression analysis, participants in the high dietary live microbe group had lower DKD prevalence than those in the low dietary live microbe group [odds ratio (OR) = 0.63, 95% confidence interval (CI), 0.50–0.78, *p* < 0.0001]. This association remained significant even after adjusting for various covariates: Model 1 [OR = 0.61, 95% CI, 0.48–0.77, *p* < 0.0001), Model 2 [OR = 0.61, 95% CI, 0.48–0.77, *p* < 0.0001), and Model 3 [OR = 0.70, 95% CI, 0.54–0.89, *p* = 0.004). However, no significant association with DKD was observed between the medium and low dietary live microbe groups in the crude model and Model 3 (*p* > 0.05).

**Table 3:** Association between different dietary live microbe groups and DKD

Character	Crude model		Model 1		Model 2		Model 3	
	OR (95% CI)	*P* value	OR (95% CI)	*P* value	OR (95% CI)	*P* value	OR (95% CI)	*P* value
Low	Ref		Ref		Ref		Ref	
Medium	0.85(0.70,1.04)	0.11	0.77(0.63,0.96)	0.02	0.78(0.63,0.96)	0.02	0.85(0.68,1.07)	0.17
High	0.63(0.50,0.78)	< 0.0001	0.61(0.48,0.77)	< 0.0001	0.61(0.48,0.77)	< 0.0001	0.70(0.54,0.89)	0.004
*P* for trend		< 0.0001		< 0.0001		< 0.0001		0.004

Model 1 adjusted for age, gender and race. Model 2 further adjusted for BMI. Model 3 further adjusted education, smoking status, PIR, UA, TG, TC, HDL-C, LDL-C, total energy, hypertension, CVD and metabolic syndrome

Data are presented as frequencies (percentages) or mean (SD)

*DKD* diabetic kidney disease, *PIR* poverty-income- ratio, *BMI* the body-mass index is determined as follows: the weight in kilograms (Kgs)/(height in square meters (m^2^), *HbA1c* haemoglobin, *Cr* serum creatinine, *UA* serum uric acid, *TG* triglycerides, *TC* total cholesterol, *HDL-C* high-density lipoprotein cholesterol, *LDL-C* low-density lipoprotein cholesterol, *eGFR* estimated glomerular filtration, *UACR* urinary albumin/creatinine ratio, *CVD* cardiovascular diseases, *CI* confidence interval, *OR* odds ratio

### Stratified analysis

**Fig. 2:**
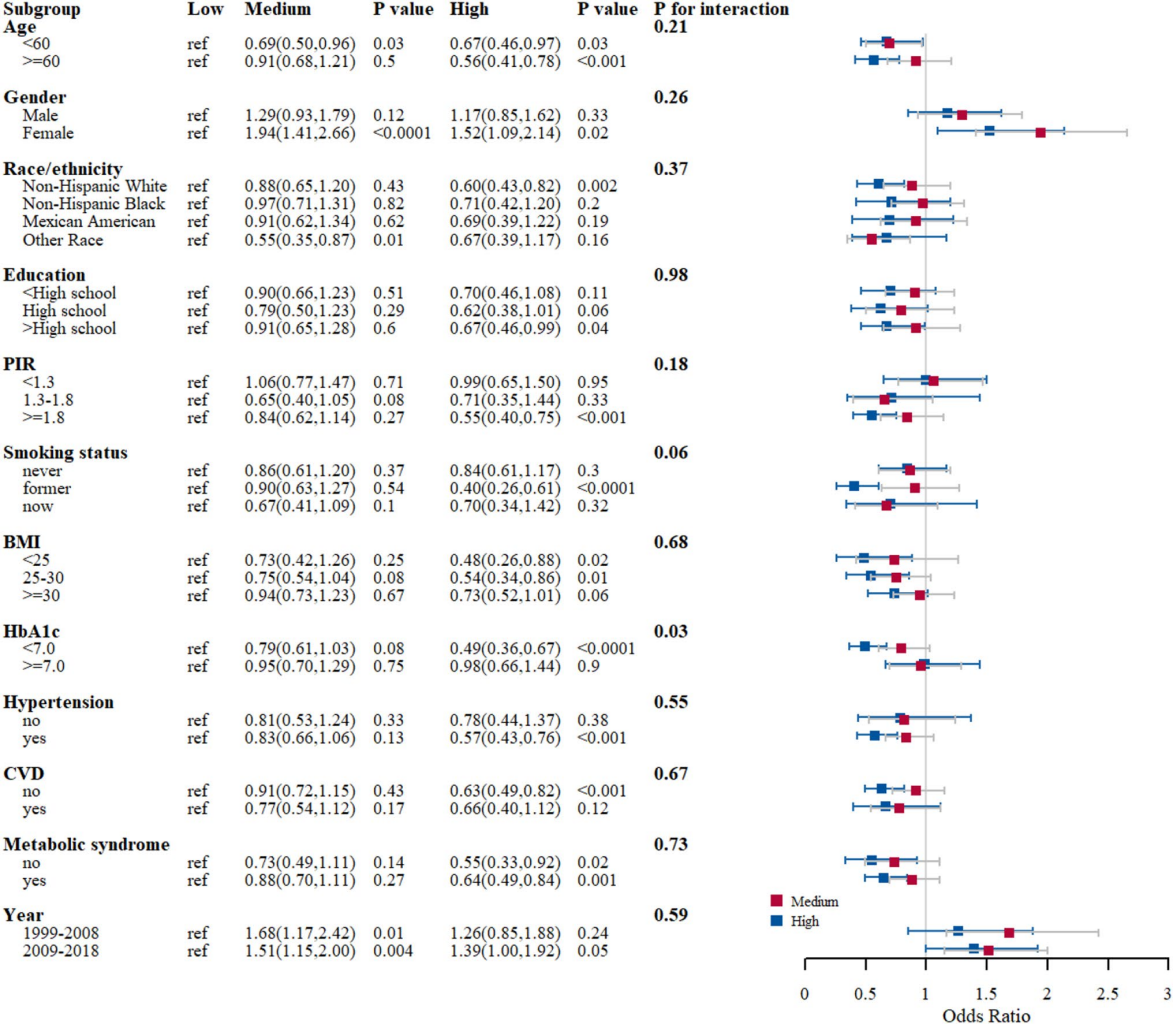
Subgroup analysis fo the association between different dietary live microbe group and DKD. Weighted univariate logistic regression was used for subgroup analysis

We performed subgroup analyses to explore the potential association between dietary live microbe groups and DKD in different populations based on age, sex, race/ethnicity, education, PIR, smoking status, BMI, HbA1c, hypertension, CVD, and metabolic syndrome. The results showed no significant differences for all predefined factors except HbA1c (*p* < 0.05, Fig. 2).

## Discussion

This study reports a novel finding in the US general population, which suggests that high dietary live microbes are negatively associated with DKD, even after adjusting for potential covariates. This finding implies that high dietary live microbes may serve as a protective factor against DKD development.

Food contains various microorganisms such as bacteria, yeasts, and molds, and the number and types of microorganisms vary depending on the food, food source, and degree of processing. Probiotics are defined as living microorganisms that can provide health benefits to the host when consumed in sufficient amounts [25]. The human diet contains various products that are sources of probiotic strains, including yogurt, kefir, sauerkraut, lobster sauce, and pickles. Probiotics consist of bacteria such as *Lactobacillus, Lactococcus, Leuconostoc, Pediococcus, Propionibacterium, Bifidobacterium, Bacillus, Streptococcus, Enterococcus, Escherichia coli, and yeast (Saccharomyces).*

Previous studies have demonstrated the benefits of probiotics and prebiotic supplements for patients with CKD. For instance, Liu et al. found that patients with CKD who consumed probiotics, prebiotics, or yogurt had a lower CKD prevalence than those who did not (OR = 0.83), indicating a lower risk of developing CKD in those who consumed these products. Specifically, a 12%, 3%, and 11% risk reduction in moderate, high, and very high risk for CKD, respectively, as observed in the multivariable-adjusted model; however, the risk reduction was not statistically significant [19]. Clinical studies have suggested that probiotics consumption, specifically Lactobacillus strains, may benefit patients with CKD. One study showed a decrease of > 10% in blood urea concentrations in patients with stage 3 or stage 4 CKD when treated with *Lactobacillus* at 16 billion CFU/day for 2 months, which was statistically significant compared to baseline measurements [26]. Another multicenter trial in patients with stage 3 or stage 4 CKD showed that uremic toxins (urea, UA, and Cr) were reduced when patients were treated with a formulation containing 90 billion CFU/day [27]. However, uremic toxins did not reach statistical significance in patients with end-stage renal disease with the administration of renadyl at 180 billion CFUs/g for just 2 months [28]. Additionally, in patients with end-stage renal disease on dialysis, administration of Lactobacillus acidophilus improved blood dimethylamine and nitrodimethylamine levels [29], as well as lowered dimethylamine and nitrosodimethylamine levels, which are known carcinogens [30]. Fundamentally, prebiotic or symbiotic supplements can modulate imbalanced gut microbiota in patients with CKD, leading to improved integrity of the intestinal epithelial barrier, decreased production of uremic toxins, and attenuation of local and systemic inflammation. Moreover, probiotics have been found to translocate harmful gut microbiome bacteria and their products, such as trimethylamine N-oxide, p-cresol, and indoxyl sulfate, which have been implicated in damaging podocytes and renal tubules through complex mechanisms [31–33]. SCFAs such as acetate, propionate, and butyrate are important metabolites produced by probiotics during the fermentation of dietary fibers and other complex carbohydrates in the gut. These SCFAs have been shown to have various health benefits, including potential protective effects in acute kidney injury (AKI) [34]. Studies, such as the one conducted by Oliveira et al., have shown that SCFAs can improve renal dysfunction caused by AKI by reducing local and systemic inflammation, oxidative cellular stress, cell infiltration/activation, and apoptosis [34]. SCFAs have also been found to promote mitochondrial biogenesis and reduce hypoxia in kidney epithelial cells, improving outcomes in AKI. These findings suggest that prebiotics, symbiotics, and probiotics may benefit CKD management, including DKD, and improve renal health by targeting the gut microbiota and its associated metabolites. Overall, the relationship between the gut microbiota and DKD involves multiple mechanisms and pathways, suggesting that further mechanistic studies are needed to determine dietary microbial benefits.

Consuming foods that provide a dietary intake of live microorganisms is beneficial in preventing DKD in adults. However, probiotics are not for everyone, and they are contraindicated in several patients (cancer, autoimmune diseases, the elderly). Despite their potential benefits, probiotics should also be used with caution in immunocompromised patients, as they may lead to infections or colonisation by pathogenic bacteria [35–37]. Further evidence is needed to determine if probiotics provide significant benefits for cancer patients, patients with autoimmune diseases, transplant patients, and the elderly.

To the best of our knowledge, this is the first comprehensive large-scale epidemiological analysis to explore the relationship between dietary live microbes (but not certain fermented foods or diets) and DKD prevalence. However, this study has several limitations. First, owing to its retrospective design, this study cannot construct or confirm causality. A large prospective cohort study should be conducted in the future to investigate the causal relationship. Second, despite adjustment for potential confounders, there may still be residual confounders that may affect the relationship between dietary live microbes and DKD. Third, recall bias may exist because respondents' dietary consumption of live microorganisms was evaluated by self-report. Fourth, participants were initially categorized into the low dietary microbe (all foods were in the low live microorganism category), medium dietary microbe (any food was in the medium live microorganism category but not high), and high dietary microbe groups (any food was in the high live microorganism category). This simple classification without precise calculation is prone to error, and the precise detection and calculation of daily dietary viable microorganisms need to be further explored. Moreover, the optimal dose of live microorganisms and its effect on DKD in different disease states needs further study. In addition, medication information for patients with DKD may not be available in NHANES, so the results needed to be interpreted with caution. Furthermore, since the population observed in this study is American, excluding special populations such as minors, we cannot analyze special populations or other races due to the limited sample size. Therefore, further studies are needed to determine whether the benefits of dietary live microbes can be extended to different populations.

In summary, this cross-sectional study, based on four cycles (1999–2018) of the NHANES database adjusted for potential confounders, shows a negative association between high dietary microbial levels and DKD in US adults. This study provides new avenues for exploring factors influencing dietary interventions to reduce DKD prevalence. In the future, there is an urgent need for more randomized controlled trials or cohort studies to confirm this finding and provide more accurate and effective prevention and treatment options for DKD prevention.

## Data Availability

The original contribution presented in this study is included in the article. The dataset was based on NHANES, which was publicly available and could be found below: https://www.cdc.gov/nchs/nhanes/.
